# Supplementary material for: Protein Succinylation and Malonylation as Potential Biomarkers in Schizophrenia
Source: J Pers Med. 2022 Aug 30;12(9):1408. doi: 10.3390/jpm12091408 (PMC9500613; doi:10.3390/jpm12091408)
Supplement: Supplementary file 1 [file jpm-12-01408-s001.zip › jpm-1827400-supplementary.pdf]

Supplementary Data S1: Patient information from postmortem donations

Supplementary Data S2: Postmortem Tissue Modification Count Tables

Supplementary Data S3: Tissue Pathway Identification Information

Supplementary Data S4: MK-801 Pathway Tables

Supplementary Data S5: Haloperidol Pathway Tables

Supplementary Data S6: Chlorpromazine Pathway Tables

Supplementary Data S7: Quetiapine Pathway Tables

Supplementary Data S8: Number of Modified Acylation Sites by MK-801 and Antipsychotics

Supplementary Data S9: Number of Succinylation and Malonylation Sites with Antipsychotic Treatment

Supplementary Data S1: Patient information from postmortem donations

Age	Gender	PMI (hours)	pH	Duration of Disease (years)	Duration of Medication (years)	Atyp typ	CPE last dose	CPE last ten years	Cause of Death	Age at Onset	Last Medication	Cigarettes	Hosp	ECT
64	F	11	6.7	48	45	3	1536	7.7	pulmonary insufficiency	16	Clozapine 500 mg, Haloperidol 40 mg, Ciatyl 40 mg	0	21	yes
73	M	20	6.6	43	40	1	507.4	1.7	heart infarction	30	Perphenazine 32 mg, Promethazine 150 mg	30/day	33	no
43	M	18	6.9	22	20	2	464	2.6	heart infarction	20	Zuclopethixol 40 mg, Valproate 1200 mg, Tiapride 300 mg	0	13	no
77	F	32	6.5	49	48	2	2555	8.3	lung embolism	28	Clozapine 400 mg, Benperidol 25 mg, Chlorprothixen 150 mg	0	48	yes
76	F	17	6.8	49	47	1	300	4.9	cardio-pulmonary insufficiency	27	Perazine 300 mg	0	30	yes
63	F	31	6.8	40	30	3	75	1.8	heart infarction	24	Olanzapine 15 mg	30/day	30	yes
92	F	37	6.9	51	48	1	100	3.4	cardio-pulmonary insufficiency	41	Prothipendyl 160 mg, Perazine 100 mg	0	51	no
71	M	28	6.4	40	35	1	782.4	10	heart infarction	30	Haloperidol 32 mg, Pipamperone 40 mg	40/day	12	no
51	M	7	6.1	25	25	1	147	0.6	heart infarction	19	Flupenthixol 15 mg	30/day	20	no
51	M	12	6.7	28	25	2	450	1.8	heart infarction	23	Clozapine 500 mg	30/day	17	no
81	M	4	6.7	62	50	1	92.8	1.4	heart insufficiency	19	Haloperidol 40 mg, Prothypendyl 80 mg	20	48	no
64	F	23	6.6	41	40	2	54.5	4.6	heart infarction	24	Zotepine 150 mg, Olanzapine 10 mg	20/day	5	yes

Table S1-Donor Information for schizophrenia postmortem tissue. No patients reported alcohol use. PMI – postmortem interval; atyptyp – relation between duration of atypical and typical neuroleptics during the patient's lifetime; CPE – calculated chlorpromazine equivalent dose (last dose, mg; last 10 years, kg); Hosp – lifetime hospitalization time (years); ECT – electroconvulsive therapy.

Age	Gender	PMI (hours)	pH	Cause of Death
41	M	7	6.5	heart infarction
91	F	16	6.7	cardio-pulmonary insufficiency
69	F	96	6.4	lung embolism
57	M	24	6.9	heart infarction
53	M	18	7.0	heart infarction
63	M	13	6.5	heart infarction
66	M	16	6.8	heart infarction
79	M	24	6.4	heart infarction

Table S2-Information for postmortem controls. No control donors reported cigarette or alcohol use. PMI – postmortem interval.

Supplementary Data S2: Postmortem Tissue Modification Count Tables

Table S3-Numbers of proteins/sites of succinylation found to be dysregulated in schizophrenia in mitochondria-enriched samples (ANOVA ≤ 0.05).

Succinylation	PCC	CN	CER	Total
Upregulated	2 / 2	2 / 2	2 / 2	6 / 6
Downregulated	8 / 7	2 / 2	6 / 6	16 / 15
Total Sites	10 / 9	4 / 4	8 / 8	22 / 21

Table S4- Numbers of proteins/sites of malonylation found to be dysregulated in schizophrenia in mitochondria-enriched samples (ANOVA ≤ 0.05).

Malonylation	PCC	CN	CER	Total
Upregulated	9 / 7	1 / 1	3 / 3	13 / 11
Downregulated	4 / 4	0 / 0	6 / 5	10 / 9
Total Sites	13 / 10	1 / 1	9 / 8	23 / 19

Table S5-Numbers of proteins/sites of succinylation found to be dysregulated in schizophrenia in total cell lysate samples (ANOVA ≤ 0.05).

Succinylation	CC	CER	Total
Upregulated	6 / 4	21 / 20	27 / 24
Downregulated	76 / 60	7 / 7	83 / 67
Total Sites	82 / 63	28 / 27	110 / 100

Table S6-Numbers of proteins/sites of malonylation found to be dysregulated in schizophrenia in total cell lysate samples (ANOVA ≤ 0.05).

Malonylation	CC	CER	Total
Upregulated	4 / 4	31 / 27	35 / 30
Downregulated	79 / 58	8 / 8	87 / 66
Total Sites	83 / 61	39 / 34	122 / 93

For all tables above: PCC, posterior cingulate cortex; CN, caudate nucleus; CER, cerebellum; CC, corpus callosum.

Supplementary Data S3: Tissue Pathway Identification Information

The conditions that are not detailed below did not identify any pathways in the Reactome Knowledgebase with an FDR ≤ 0.01 and are not included here.

Table S7-Pathways identified with Reactome.org with FDR ≤ 0.01 for proteins with downregulated sites of succinylation in mitochondria-enriched lysates.

Downregulated Succinylation Mitochondria	Entities				Reactions	
Pathway Name	Found	Ratio	p-Value	FDR	Found	Ratio
Detoxification of Reactive Oxygen Species	4 / 39	0.003	5.57e-07	2.75e-04	5 / 34	0.003

Table S8-Pathways identified with Reactome.org with FDR ≤ 0.01 for proteins with up- and down-regulated sites of succinylation in mitochondria-enriched lysates.

Overall Succinylation Mitochondria	Entities				Reactions	
Pathway Name	Found	Ratio	p-Value	FDR	Found	Ratio
Detoxification of Reactive Oxygen Species	4 / 39	0.003	1.79e-06	9.48e-04	5 / 34	0.003
Cellular responses to stress	7 / 408	0.037	2.36e-05	0.006	16 / 184	0.015

Table S9-Pathways identified with Reactome.org with FDR ≤ 0.01 for proteins with downregulated sites of succinylation in total cell lysates.

Downregulated Succinylation Total Lysate	Entities				Reactions	
Pathway Name	Found	Ratio	p-Value	FDR	Found	Ratio
Regulation of HSF1-mediated heat shock response	6 / 75	0.007	5.97e-06	0.002	7 / 14	0.001
Cellular response to heat stress	6 / 95	0.009	2.25e-05	0.004	11 / 29	0.002
trans-Golgi Network Vesicle Budding	5 / 74	0.007	8.24e-05	0.01	9 / 19	0.002

Table S10-Pathways identified with Reactome.org with FDR ≤ 0.01 for proteins with up- and down-regulated sites of succinylation in total cell lysates.

Overall Succinylation Total Lysate	Entities				Reactions	
Pathway Name	Found	Ratio	p-Value	FDR	Found	Ratio
Regulation of HSF1-mediated heat shock response	7 / 75	0.007	2.46e-06	0.001	7 / 14	0.001
Cristae formation	5 / 31	0.003	5.38e-06	0.001	2 / 2	1.65e-04
Cellular response to heat stress	7 / 95	0.009	1.14e-05	0.001	11 / 29	0.002
Mitochondrial biogenesis	7 / 96	0.009	1.21e-05	0.001	5 / 36	0.003
Formation of ATP by chemiosmotic coupling	4 / 18	0.002	1.42e-05	0.001	3 / 3	2.48e-04

Table S11-Pathways identified with Reactome.org with FDR ≤ 0.01 for proteins with downregulated sites of malonylation in total cell lysates.

Downregulated Malonylation Total Lysate	Entities				Reactions	
Pathway Name	Found	Ratio	p-Value	FDR	Found	Ratio
COPI-mediated anterograde transport	8 / 102	0.009	2.50e-07	4.98e-05	6 / 12	9.91e-04
ER to Golgi Anterograde Transport	9 / 155	0.015	5.20e-07	5.14e-05	17 / 39	0.003
Transport to the Golgi and subsequent modification	9 / 186	0.017	2.31e-06	1.82e-04	17 / 60	0.005
Membrane Trafficking	15 / 635	0.057	6.46e-06	3.99e-04	73 / 218	0.018
Neutrophil degranulation	13 / 480	0.043	7.00e-06	3.99e-04	7 / 10	8.25e-04
COPI-independent Golgi-to-ER retrograde traffic	5 / 53	0.005	2.12e-05	0.001	2 / 7	5.78e-04
HSP90 chaperone cycle for steroid hormone receptors (SHR)	5 / 57	0.005	3.00e-05	0.001	1 / 12	9.91e-04
Vesicle-mediated transport	15 / 761	0.068	5.36e-05	0.002	73 / 251	0.021
Caspase-mediated cleavage of cytoskeletal proteins	3 / 12	0.001	6.20e-05	0.002	3 / 10	8.25e-04
Organelle biogenesis and maintenance	9 / 298	0.027	9.29e-05	0.003	19 / 86	0.007
Asparagine N-linked glycosylation	9 / 305	0.027	1.11e-04	0.003	17 / 144	0.012
Cilium Assembly	7 / 202	0.018	2.59e-04	0.007	17 / 50	0.004

Table S12-Pathways identified with Reactome.org with FDR ≤ 0.01 for proteins with up- and down-regulated sites of malonylation in total cell lysates.

Overall Malonylation Total Lysate	Entities				Reactions	
Pathway Name	Found	Ratio	p-Value	FDR	Found	Ratio
COPI-mediated anterograde transport	10 / 107	0.008	3.09E-09	8.53E-07	6 / 12	9.91E-04
ER to Golgi Anterograde Transport	11 / 164	0.012	1.44E-08	1.99E-06	17 / 39	0.003
Transport to the Golgi and subsequent modification	11 / 219	0.015	2.58E-07	2.84E-05	17 / 60	0.005
Membrane Trafficking	18 / 665	0.047	3.59E-07	3.30E-05	73 / 218	0.018
Vesicle-mediated transport	19 / 824	0.058	1.78E-06	1.39E-04	77 / 251	0.021
COPI-independent Golgi-to-ER retrograde traffic	6 / 63	0.004	4.53E-06	3.12E-04	2 / 7	5.78E-04
HSP90 chaperone cycle for steroid hormone receptors (SHR)	6 / 70	0.005	8.21E-06	5.01E-04	1 / 12	9.91E-04
Neutrophil degranulation	13 / 480	0.034	1.80E-05	9.92E-04	7 / 10	8.25E-04
Asparagine N-linked glycosylation	12 / 421	0.030	2.37E-05	0.001	20 / 144	0.012
Recruitment of NuMA to mitotic centrosomes	6 / 97	0.007	5.04E-05	0.002	2 / 2	1.65E-04
Interaction between L1 and Ankyrins	4 / 33	0.002	7.62E-05	0.003	2 / 4	3.30E-04
Caspase-mediated cleavage of cytoskeletal proteins	3 / 12	0.001	7.71E-05	0.003	3 / 10	8.25E-04
Cilium Assembly	8 / 208	0.015	7.88E-05	0.003	17 / 50	0.004
Organelle biogenesis and maintenance	10 / 335	0.024	8.14E-05	0.003	19 / 86	0.007
Signaling by Rho GTPases	11 / 457	0.032	2.32E-04	0.007	15 / 117	0.010
L1CAM interactions	6 / 130	0.009	2.45E-04	0.007	4 / 54	0.004
Apoptosis	7 / 187	0.013	2.64E-04	0.008	8 / 139	0.011
Programmed Cell Death	7 / 195	0.014	3.39E-04	0.009	8 / 152	0.013

Supplementary Data S4: MK-801 Pathway Tables

Table S13-Top 10 upregulated succinylation pathways in MK-801-treated cells from Reactome.org.

Category	Pathway Name
Various	Metabolism of RNA
Various	Translation
Semaphorin / ROBO	Axon guidance
Ceruloplasmin	L13a-mediated translational silencing of Ceruloplasmin expression
Elongation Factors	GTP hydrolysis and joining of the 60S ribosomal subunit
Ceruloplasmin / Elongation Factors	Cap-dependent Translation Initiation
	Eukaryotic Translation Initiation
AUF1 & HuR (ELAVL1 / YWHAZ)	Regulation of mRNA stability by proteins that bind AU-rich elements
AUF1	AUF1 (hnRNP D0) binds and destabilizes mRNA
ROBO	Signaling by ROBO receptors

37 of 196 accession numbers were not found in the Reactome database.

Table S14-Top 10 downregulated succinylation pathways in MK-801-treated cells from Reactome.org.

Category	Pathway Name
Intron Processing / NMD	Metabolism of RNA
Elongation Factors	Eukaryotic Translation Elongation
Spliceosomes / hnRNPs	mRNA Splicing - Major Pathway
	mRNA Splicing
ROBO	Signaling by ROBO receptors
	Regulation of expression of SLITs and ROBOs
Various	Translation
NMD / Stress Response	Nonsense-Mediated Decay (NMD)
	Nonsense Mediated Decay (NMD) enhanced by the Exon Junction Complex (EJC)
hnRNPs	Processing of Capped Intron-Containing Pre-mRNA

37 of 190 accession numbers were not found in the Reactome database.

Table S15-Top 10 upregulated malonylation pathways in MK-801-treated cells from Reactome.org.

Category	Pathway Name
CCT/TriC	Prefoldin mediated transfer of substrate to CCT/TriC
	Folding of actin by CCT/TriC
	Cooperation of Prefoldin and TriC/CCT in actin and tubulin folding
SRP	SRP-dependent cotranslational protein targeting to membrane
Various	Metabolism of RNA
ROBO	Axon guidance
ROBO	Regulation of expression of SLITs and ROBOs
CCT/TriC	Formation of tubulin folding intermediates by CCT/TriC
Ceruloplasmin	L13a-mediated translational silencing of Ceruloplasmin expression
Various	Translation

32 of 150 accession numbers were not found in the Reactome database.

Table S16-Top 10 downregulated malonylation pathways in MK-801-treated cells from Reactome.org. 31 of 140 accession numbers were not found in the Reactome database.

Category	Pathway Name
	Folding of actin by CCT/TriC
	Prefoldin mediated transfer of substrate to CCT/TriC
CCT/TriC	Cooperation of Prefoldin and TriC/CCT in actin and tubulin folding
	Formation of tubulin folding intermediates by CCT/TriC
	Association of TriC/CCT with target proteins during biosynthesis
HSF1	Cellular response to heat stress
CCT/TriC	Chaperonin-mediated protein folding
	Protein folding
Various	Metabolism of RNA
HSP90	HSP90 chaperone cycle for steroid hormone receptors (SHR)

Table S17-Top 10 affected overall succinylation pathways in MK-801-treated cells from Reactome.org. 65 of 322 accession numbers were not found in the Reactome database.

Category	Pathway Name
Various	Metabolism of RNA
Various	Translation
mRNA Processing	mRNA Splicing - Major Pathway
mRNA Processing	mRNA Splicing
Translation	GTP hydrolysis and joining of the 60S ribosomal subunit
ROBO	Regulation of expression of SLITs and ROBOs
Translation	Eukaryotic Translation Elongation
Translation	Cap-dependent Translation Initiation
Translation	Eukaryotic Translation Initiation
Ceruloplasmin	L13a-mediated translational silencing of Ceruloplasmin expression

Table S18-Top 10 affected overall malonylation pathways in MK-801-treated cells from Reactome.org. 52 of 247 accession numbers were not found in the Reactome database.

Category	Pathway Name
CCT/TriC	Folding of actin by CCT/TriC
Various	Metabolism of RNA
CCT/TriC	Prefoldin mediated transfer of substrate to CCT/TriC
CCT/TriC	Cooperation of Prefoldin and TriC/CCT in actin and tubulin folding
CCT/TriC	Formation of tubulin folding intermediates by CCT/TriC
Translation	Eukaryotic Translation Elongation
Translation	Peptide chain elongation
Ceruloplasmin	L13a-mediated translational silencing of Ceruloplasmin expression
Protein Trafficking	SRP-dependent cotranslational protein targeting to membrane
Translation	GTP hydrolysis and joining of the 60S ribosomal subunit

Supplementary Data S5: Haloperidol Pathway Tables

Table S19-Upregulated succinylated pathways by haloperidol in MO3.13 cells from Reactome.org. 46 of 230 accession numbers were not found in the Reactome database.

Category	Pathway Name
rRNA/mRNA Processing and NMD	Metabolism of RNA
Ceruloplasmin	L13a-mediated translational silencing of Ceruloplasmin expression
ROBO	Regulation of expression of SLITs and ROBOs
NMD	Nonsense-Mediated Decay (NMD)
NMD	Nonsense Mediated Decay (NMD) enhanced by the Exon Junction Complex (EJC)
Translation	Cap-dependent Translation Initiation
Translation	Eukaryotic Translation Initiation
ROBO	Signaling by ROBO receptors
Translation	GTP hydrolysis and joining of the 60S ribosomal subunit
Translation	Formation of a pool of free 40S subunits

Table S20-Downregulated succinylated pathways by haloperidol in MO3.13 cells from Reactome.org. 35 of 169 accession numbers were not found in the Reactome database.

Category	Pathway Name
Various	Translation
Various	RNA Metabolism
ROBO	Axon guidance
Translation	Eukaryotic Translation Elongation
Stress Response	Cellular responses to stress
Translation	Cytosolic tRNA aminoacylation
Translation	rRNA processing
Translation	Major pathway of rRNA processing in the nucleolus and cytosol
Vesicles	Influenza Life Cycle
ROBO	Signaling by ROBO receptors

Table S21-Upregulated malonylated pathways by haloperidol in MO3.13 cells from Reactome.org. 42 of 210 accession numbers were not found in the Reactome database.

Category	Pathway Name
Translation	Peptide chain elongation
Translation	Translation
Translation	Eukaryotic Translation Elongation
Ceruloplasmin	L13a-mediated translational silencing of Ceruloplasmin expression
Ribosomal Function	GTP hydrolysis and joining of the 60S ribosomal subunit
Ribosomal Function	Formation of a pool of free 40S subunits
Translation	Cap-dependent Translation Initiation
Translation	Eukaryotic Translation Initiation
NMD	Nonsense Mediated Decay (NMD) independent of the Exon Junction Complex (EJC)
Protein Trafficking	SRP-dependent cotranslational protein targeting to membrane

Table S22-Downregulated malonylated pathways by haloperidol in MO3.13 cells from Reactome.org. 23 of 126 accession numbers were not found in the Reactome database.

Category	Pathway Name
Translation	Eukaryotic Translation Elongation
Translation	Peptide chain elongation
RNA Metabolism	Metabolism of RNA
Infection	Influenza Life Cycle
Infection	Influenza Infection
Translation	Translation
Translation	Formation of a pool of free 40S subunits
Vesicles	Infectious disease
RNA Metabolism	rRNA processing
Ceruloplasmin	L13a-mediated translational silencing of Ceruloplasmin expression

Supplementary Data S6: Chlorpromazine Pathway Tables

Table S23-Upregulated succinylated pathways by chlorpromazine in MO3.13 cells from Reactome.org. 15 of 95 accession numbers were not found in the Reactome database.

Category	Pathway Name
Stress Response	Cellular responses to stress
Stress Response	Cellular response to heat stress
Stress Response	Cellular responses to external stimuli
Stress Response	Regulation of HSF1-mediated heat shock response
RNA Metabolism	Metabolism of RNA
Ceruloplasmin	L13a-mediated translational silencing of Ceruloplasmin expression
Translation	GTP hydrolysis and joining of the 60S ribosomal subunit
Translation	Translation
Translation	Cap-dependent Translation Initiation
Translation	Eukaryotic Translation Initiation

Table S24-Downregulated succinylated pathways by chlorpromazine in MO3.13 cells from Reactome.org. 11 of 70 accession numbers were not found in the Reactome database.

Category	Pathway Name
Ceruloplasmin	L13a-mediated translational silencing of Ceruloplasmin expression
Translation	Cap-dependent Translation Initiation
Translation	Eukaryotic Translation Initiation
Translation	Translation
Translation	GTP hydrolysis and joining of the 60S ribosomal subunit
NMD	Nonsense Mediated Decay (NMD) independent of the Exon Junction Complex (EJC)
Translation	Formation of a pool of free 40S subunits
Vesicles	Influenza Infection
Protein Trafficking	SRP-dependent cotranslational protein targeting to membrane
ROBO	Regulation of expression of SLITs and ROBOs

Table S25-Overall dysregulated succinylated pathways by chlorpromazine in MO3.13 cells from Reactome.org. 25 of 151 accession numbers were not found in the Reactome database.

Category	Pathway Name
Ceruloplasmin	L13a-mediated translational silencing of Ceruloplasmin expression
Translation	Cap-dependent Translation Initiation
Translation	Eukaryotic Translation Initiation
Translation	Translation
Translation	GTP hydrolysis and joining of the 60S ribosomal subunit
ROBO	Regulation of expression of SLITs and ROBOs
RNA Metabolism	Metabolism of RNA
Translation	Peptide chain elongation
Translation	Eukaryotic Translation Elongation
Translation	Formation of a pool of free 40S subunits

Table S26-Upregulated malonylated pathways by chlorpromazine in MO3.13 cells from Reactome.org. 18 of 98 accession numbers were not found in the Reactome database.

Category	Pathway Name
Stress Response	HSP90 chaperone cycle for steroid hormone receptors (SHR)
Translation	L13a-mediated translational silencing of Ceruloplasmin expression
Translation	GTP hydrolysis and joining of the 60S ribosomal subunit
Translation	Cap-dependent Translation Initiation
Translation	Eukaryotic Translation Initiation
Cytoskeleton	Axon guidance
Translation	Translation initiation complex formation
Translation	Ribosomal scanning and start codon recognition
Translation	Activation of the mRNA upon binding of the cap-binding complex and eIFs, and subsequent binding to 43S
Cytoskeleton	Formation of tubulin folding intermediates by CCT/TriC

Table S27-Downregulated malonylated pathways by chlorpromazine in MO3.13 cells from Reactome.org. 9 of 65 accession numbers were not found in the Reactome database.

Category	Pathway Name
RNA Metabolism	Metabolism of RNA
Protein Trafficking	SRP-dependent cotranslational protein targeting to membrane
NMD	Nonsense-Mediated Decay (NMD)
NMD	Nonsense Mediated Decay (NMD) enhanced by the Exon Junction Complex (EJC)
RNA Metabolism	Selenoamino acid metabolism
Translation	Peptide chain elongation
Vesicles	Influenza Infection
Translation	Eukaryotic Translation Termination
RNA Metabolism	Selenocysteine synthesis
Translation	Eukaryotic Translation Elongation

Table S28-Overall dysregulated malonylated pathways by chlorpromazine in MO3.13 cells from Reactome.org. 26 of 154 accession numbers were not found in the Reactome database.

Category	Pathway Name
Translation	L13a-mediated translational silencing of Ceruloplasmin expression
Translation	GTP hydrolysis and joining of the 60S ribosomal subunit
RNA Metabolism	Metabolism of RNA
Translation	Cap-dependent Translation Initiation
Translation	Eukaryotic Translation Initiation
NMD	Nonsense-Mediated Decay (NMD)
NMD	Nonsense Mediated Decay (NMD) enhanced by the Exon Junction Complex (EJC)
Translation	Translation initiation complex formation
Translation	Ribosomal scanning and start codon recognition
Translation	Activation of the mRNA upon binding of the cap-binding complex and eIFs, and subsequent binding to 43S

Supplementary Data S7: Quetiapine Pathway Tables

Table S29-Upregulated succinylated pathways by quetiapine in MO3.13 cells from Reactome.org. 12 of 54 accession numbers were not found in the Reactome database.

Category	Pathway Name
Lamin Dimers	Depolymerisation of the Nuclear Lamina
Stress Response	Unfolded Protein Response (UPR)
RNA Metabolism	Metabolism of RNA
Cell Cycle	Nuclear Envelope Breakdown
RNA Metabolism	Processing of Capped Intron-Containing Pre-mRNA
RNA Metabolism	mRNA Splicing - Major Pathway
RNA Metabolism	Major pathway of rRNA processing in the nucleolus and cytosol
RNA Metabolism	mRNA Splicing
RNA Metabolism	rRNA processing in the nucleus and cytosol
RNA Metabolism	rRNA processing

Downregulated succinylated pathways by quetiapine in MO3.13 cells did not exhibit an FDR below 4% and were not included in this analysis.

Table S30-Overall dysregulated succinylated pathways by quetiapine in MO3.13 cells from Reactome.org. 12 of 57 accession numbers were not found in the Reactome database.

Category	Pathway Name
RNA Metabolism	Metabolism of RNA
RNA Metabolism	Major pathway of rRNA processing in the nucleolus and cytosol
Lamin Dimers	Depolymerisation of the Nuclear Lamina
RNA Metabolism	rRNA processing
Stress Response	Unfolded Protein Response (UPR)
Cell Cycle	Nuclear Envelope Breakdown
RNA Metabolism	Processing of Capped Intron-Containing Pre-mRNA
RNA Splicing	mRNA Splicing - Major Pathway
Protein Metabolism	Metabolism of proteins
RNA Metabolism	mRNA Splicing

Table S31-Upregulated malonylated pathways by quetiapine in MO3.13 cells from Reactome.org. 9 of 47 accession numbers were not found in the Reactome database.

Category	Pathway Name
RNA Metabolism	Metabolism of RNA
Translation	GTP hydrolysis and joining of the 60S ribosomal subunit
Translation	Eukaryotic Translation Initiation
Translation	Cap-dependent Translation Initiation
Ceruloplasmin	L13a-mediated translational silencing of Ceruloplasmin expression
RNA Metabolism	rRNA processing
NMD	Nonsense Mediated Decay (NMD) independent of the Exon Junction Complex (EJC)
ROBO	Regulation of expression of SLITs and ROBOs
RNA Metabolism	Major pathway of rRNA processing in the nucleolus and cytosol
RNA Metabolism	rRNA processing in the nucleus and cytosol

Table S32-Downregulated malonylated pathways by quetiapine in MO3.13 cells from Reactome.org. 1 of 5 accession numbers were not found in the Reactome database.

Category	Pathway Name
Vesicles	Lysosome Vesicle Biogenesis
Cell Receptors	HSP90 chaperone cycle for steroid hormone receptors (SHR)
Vesicles	Clathrin derived vesicle budding
Vesicles	trans-Golgi Network Vesicle Budding
Clathrin (Vesicles)	MHC class II antigen presentation
Heat Shock HSP90	Aryl hydrocarbon receptor signalling
Heat Shock HSP90	Uptake and function of diphtheria toxin
Gap Junctions	Formation of annular gap junctions
Clathrin (Vesicles)	Entry of Influenza Virion into Host Cell via Endocytosis
Gap Junctions	Gap junction degradation

Table S33-Overall dysregulated malonylated pathways by quetiapine in MO3.13 cells from Reactome.org. 10 of 52 accession numbers were not found in the Reactome database.

Category	Pathway Name
RNA Metabolism	Metabolism of RNA
Translation	GTP hydrolysis and joining of the 60S ribosomal subunit
Translation	Eukaryotic Translation Initiation
Translation	Cap-dependent Translation Initiation
Ceruloplasmin	L13a-mediated translational silencing of Ceruloplasmin expression
ROBO	Axon guidance
RNA Metabolism	rRNA processing
Vesicles	Lysosome Vesicle Biogenesis
Macromolecule Export/Clathrin	Influenza Life Cycle
NMD	Nonsense Mediated Decay (NMD) independent of the Exon Junction Complex (EJC)

Supplementary Data S8: Total Number of Modified Acylation Sites by MK-801 and Antipsychotics

Table S34-Sites of protein acylation in MK-801-treated cells. Classified by Uniprot Accession Number (AN).

	Malonylation	Succinylation
Total Sites	349	502
Unique ANs	247	322
ANs Upregulated	150	196
ANs Downregulated	140	190
Both Up- and Down-regulated	43	64

Supplementary Data S9: Number of Succinylation and Malonylation Sites with Antipsychotic Treatment

Table S35-Numbers of statistically perturbed (ANOVA ≤ 0.05) succinylation and malonylation sites in MO3.13 cells due to antipsychotic incubation.

Compound	Haloperidol		Chlorpromazine		Quetiapine	
Modification	SuccK	MalK	SuccK	MalK	SuccK	MalK
Overall Number of Sites	538	434	189	186	61	55
Percentage of Modified Peptides	35.8%	34.1%	20.6%	20.2%	3.51%	3.51%
Percentage of Total Peptides	3.16%	2.31%	1.15%	1.41%	0.21%	0.21%
